# The Real Value of the Medicinal Peroxide of Hydrogen Preparations Found in the Market

**Published:** 1895-04

**Authors:** H. Endemann

**Affiliations:** Chemist; Formerly Associate Chemist to the New York City Board of Health


### THE REAL VALUE OF THE MEDICINAL PEROXIDE OF HYDROGEN PREPARATIONS FOUND IN THE MARKET.

BY H. ENDEMANN, PH. D., CHEMIST.
Formerly Associate Chemist to the New York City Board of Health.
(*Abstract from the Times and Register, of Philadelphia, Pa., December* 15, 1894.)

In this valuable article, the writer states that a standard solution of medicinal H2 O2 must answer the following tests:

1. It should contain at least 15 volumes of available oxygen.
2. The quantity of free acids contained in 100 cubic centimetres, should require not less than 1 c. c., and not more than 3 c. c. of normal volumetric soda solution, to be made neutral. Such a small quantity of free acid is not objectionable.
3. It should not contain any soluble baryta salts.
4. It must be free from sediment.

The different brands which he found on the market, being submitted to the above tests, gave the following results:

| BRANDS OF $H_2O_2$ SOLUTIONS. | | Volume of Available Oxygen determined by means of a solution containing 5.665 Grammes of Permanganate of Potash per liter of distilled water. | Residue obtained from 100 C. C. of Peroxide of Hydrogen dried at 120 degrees C. | Acidity expressed in Cubic centimeters of Normal Volumetric Soda Solution for 100 C. C. of Peroxide. | Baryta found in Soluble Baryta Salts contained in 100 C. C. of Peroxide. |
|---|---|---|---|---|---|
| No. 1. John Bene's | $H_2O_2$ (Medicinal) | 10.50 | 0.1886 | 2.19 | None |
| No. 2. Hydrozone | ................ | 27.35 | 0.2180 | 3.11 | " |
| No. 3. Larkin & Scheffer's | $H_2O_2$ (Medicinal) | 9.65 | 0.1206 | 6.75 | " |
| No. 4. Mallinckrodt's | " " | 9.55 | 0.1408 | 1.43 | " |
| No. 5. Marchand's | " " | 16.55 | 0.564 | 1.29 | " |
| No. 6. McKesson & Robbins' | " " | 10.95 | 0.0540 | 0.44 | " |
| No. 7. Merck & Co.'s | " " | 0.50 | 0.2418 | 4.57 | " |
| No. 8. Oakland Chemical Co.'s | " " | 10.50 | 0.0382 | 0.34 | 0.0017 |
| No. 9. Peuchot's | " " | 10.60 | 0.4674 | 1.77 | 0.0018 |
| No. 10. Powers & Weightman's | " " | 8.40 | 0.0830 | 2.03 | None |
| No. 11. Pyrozone, 3 per cent. | " " | 11.20 | 0.0534 | 0.76 | " |
| No. 12. Rosengarten & Son's | " " | 3.10 | 0.1002 | 0.25 | " |
| No. 13. Smith, Kline & French Co.'s | " " | 6.15 | 0.0880 | 2.6 | " |
| No. 14. E. R. Squibb's | " " | 12.40 | 1.004 | 12.04 | " |

By referring to this table, it is easily noticed that brands No. 7 and No. 12 are valueless.

The brands No. 8 and No. 9 are not fit for medicinal uses, owing to the fact that they contain traces of soluble baryta salts.

The brand No. 3 has a heavy sediment of sulphate of baryta, which may be considered inert towards the system, but it is certainly detrimental to the keeping qualities of this preparation.

Brand No. 14, which is sold as a ten-volume solution, is really twelve volumes, but it is too acid.

Brand No. 5, which is sold as a fifteen-volume solution, is really 16.55 volumes, viz.: About 10 per cent. above the standard.

The brand No. 2, which is sold without any mention of volume, is really a 27.35-volume solution, viz.: Ninety per cent. above the standard.

None of the other brands come up to the standard, but, on the contrary, they run from 35 to 55 per cent. below.

